# Nomogram for Predicting In-Hospital Mortality in Patients with Acute ST-Elevation Myocardial Infarction Complicated by Cardiogenic Shock after Primary Percutaneous Coronary Intervention

**DOI:** 10.1155/2022/8994106

**Published:** 2022-03-12

**Authors:** Yudan Wang, Litian Liu, Xinning Li, Yi Dang, Yingxiao Li, Jiaqi Wang, Xiaoyong Qi

**Affiliations:** ^1^School of Graduate, Hebei Medical University, Shijiazhuang, Hebei Province, China; ^2^Department of Cardiology Center, Hebei General Hospital, Shijiazhuang, Hebei Province, China; ^3^School of Graduate, Hebei North University, Zhangjiakou, Hebei Province, China

## Abstract

**Background:**

Mortality after percutaneous coronary intervention (PCI) in ST-elevation myocardial infarction (STEMI) patients with cardiogenic shock (CS) remains high. However, the real-world risk factors for mortality in these patients are poorly defined.

**Objective:**

The aim of this study is to establish a clinical prognostic nomogram for predicting in-hospital mortality after primary PCI in STEMI patients with CS.

**Methods:**

This retrospective, multicenter, observational study included STEMI patients with CS who underwent PCI at 39 hospitals in Hebei Province from January 2018 to December 2019. A multivariate logistic regression model was used to identify the factors associated with in-hospital mortality. These factors were then incorporated into a nomogram and its performance was evaluated by discrimination, calibration, and clinical utility.

**Results:**

This study included 274 patients, among whom 179 died in hospital. Sex, random blood glucose on admission, ejection fraction after PCI, no-reflow, and intra-aortic balloon pump (IABP) were independently associated with in-hospital mortality (all *P* < 0.05). In the training set, the nomogram showed a C-index of 0.819, goodness-of-fit of 0.08, and area under the receiver operating characteristic curve (AUC) of 0.819 (95%CI = 0.759–0.879). In the testing set, the C-index was 0.842, goodness-of-fit was 0.585, and AUC was 0.842 (95%CI = 0.715–0.970). The results indicate that the nomogram had good discrimination and good prediction accuracy and could achieve a good net benefit.

**Conclusion:**

We established and validated a nomogram that provided individual prediction of in-hospital mortality for STEMI patients with CS after PCI in a Chinese population.

## 1. Introduction

Cardiogenic shock (CS) is typically caused by acute myocardial infarction (AMI) with subsequent left ventricular (LV) dysfunction and failure to provide sufficient cardiac output, despite a normal or elevated preload. The incidence of CS after ST-elevation myocardial infarction (STEMI) was reported to be higher than for non-STEMI (NSTEMI) (2.5%) [1]. There are numerous clinical complications that can appear with STEMI development, although CS is the most devastating and has the worst prognosis. The incidence of CS in STEMI patients is estimated as 5%–15% [2]. In the past few decades, the management of STEMI has markedly improved, mainly because of the increased use of mechanical and drug reperfusion methods and development of emerging technologies [3]. However, in the subgroup of patients with CS (CS-STEMI), improvements remain minimal. As such, CS-STEMI remains the leading cause of death, with in-hospital mortality rates approaching 50% [4–6].

The current American College of Cardiology (ACC)/American Heart Association (AHA) guidelines recommend primary percutaneous coronary intervention (PCI) in CS-STEMI patients [7]. The SHOCK (Should We Emergently Revascularize Occluded Coronaries for Cardiogenic Shock) study suggests that early revascularization must be strongly considered for patients with AMI complicated by CS. Nevertheless, even if these patients receive timely PCI and/or appropriate antiplatelet drugs, mortality remains slightly elevated and a substantial number of patients still die in-hospital after PCI. As such, the in-hospital outcomes after PCI can still be improved.

A number of studies have examined the risk factors for short-term and long-term mortality in CS-STEMI patients after PCI, which include PCI failure, a final Thrombolysis in Myocardial Infarction (TIMI) grade flow of 0–2, and multivessel disease [8, 9]. The Global Registry for Acute Coronary Events (GRACE) risk score is the most widely used tool, although the TIMI risk score is a simple alternative [10, 11]. Unfortunately, there is limited information on the predictors of in-hospital mortality in CS-STEMI patients and those receiving PCI. The aim of the present study was to establish a prediction model of in-hospital mortality risk for use shortly after admission, with the goal of providing clinical guidance and improving the outcomes of CS-STEMI patients.

## 2. Materials and Methods

### 2.1. Study Design and Patients

This multicenter, retrospective, observational study included all patients who underwent PCI for CS-STEMI in Hebei Province from January 2018 to December 2019. All patients met the diagnostic criteria of acute STEMI based on their symptoms and/or electrocardiogram, and myocardial damage markers, and underwent primary PCI according to the European Society of Cardiology (ESC) guidelines (2017) for the management of STEMI [12], These guidelines include persistent chest discomfort or other symptoms suggestive of ischemia and ST-segment elevation in at least two contiguous leads. CS is defined by one, two, or all three of the following parameters: systolic blood pressure <90 mmHg (after adequate fluid challenge) for 30 min, requires vasopressor therapy to maintain systolic blood pressure >90 mmHg, or signs of hypoperfusion (altered mental status/confusion, cold periphery, oliguria <0.5 mL/kg/h for the prior 6 h, or blood lactate >2 mmol/L) [13]. Patients with NSTEMI or unstable angina, or STEMI patients who did not undergo PCI, were excluded. Patients who were readmitted to the hospital for revascularization of noncriminal vessels were also excluded. The treatment strategy during and after PCI of the patients is determined by the doctor in charge in accordance with relevant guidelines. This study was approved by the Ethics Committees of Hebei General Hospital as the lead center and by the ethics committee of each participating hospital (ID:202183). The study was conducted according to the tenets of the Declaration of Helsinki for medical research involving human subjects and good clinical practice.

### 2.2. Data Collection

Demographics (age, sex, and body mass index (BMI)), medical history (hypertension, diabetes mellitus, atrial fibrillation, hyperlipidemia, and family history of coronary artery disease, renal failure, or peripheral artery disease), angiographic characteristics, information on cardiac procedures (disease condition, TIMI flow grade, use of an intra-aortic balloon pump (IABP), use of a temporary pacemaker, use of a ventilator, and whether there was no-reflow, coronary perforation, or cardiac arrest), medications on admission (antiplatelet agents, *β*-blockers, angiotensin-converting enzyme inhibitors, angiotensin receptor blockers, and statins), biochemical markers (Neutrophil to Lymphocyte (N/L ratio) hematocrit, hemoglobin, platelet, and random blood glucose on admission), and LV ejection fraction (LVEF) after PCI were extracted from the medical charts. All treatments were performed according to current guidelines.

### 2.3. Nomogram Construction

Demographics, medical history, vital signs before and after PCI, and auxiliary examinations were evaluated using univariate logistic regression. Variables with *P* < 0.05 in the univariate logistic analyses were included for multivariate logistic analysis and nomogram construction. The receiver operator characteristic (ROC) curve analysis was used to quantify the prediction performance of the nomogram. A calibration curve was used to evaluate the calibration of the nomogram, while its goodness-of-fit was assessed using the Hosmer–Lemeshow test. Finally, the clinical utility of the nomogram was accessed using a decision curve analysis (DCA).

### 2.4. Statistical Analysis

Statistical analyses were performed using statistical software (R v4.0.3; R Foundation for Statistical Computing; RStudio v1.3.959; RStudio, Auckland, New Zealand). The R packages used in this study were rms, reader, table one, pROC, ResourceSelection, and rmda. The predictive accuracy of the nomogram was measured using the C-statistic (bootstrap method, 1000 times). Calibration was evaluated using the Hosmer–Lemeshow statistics. Categorical variables were presented as frequencies with percentages, normally distributed continuous variables as means ± standard deviation, and other data as medians with interquartile ranges. Categorical variables were compared using the chi-square test or Fisher's test if the expected cell count was <5. The Student's *t*-test was used to compare normally distributed continuous variables. The Mann–Whitney U-test was used to compare non-normally distributed data. The significance level was set at 0.05 and two-sided tests were used.

## 3. Results

### 3.1. Characteristics of the Patients

The entire study population consisted of 274 patients diagnosed with STEMI complicated by CS and who underwent PCI, including 231 patients in the training set (155 (67.1%) deceased patients and 76 (32.9%) survivors) and 43 patients in the test set (24 (55.8%) deceased patients and 19 (44.2%) survivors) (Table 1). The clinical characteristics, including demographic, medical history, angiographic characteristics, and information of cardiac procedures, medications, and biochemical markers, are summarized in Supplementary Table 1. The clinical characteristics selected as predictors for the nomogram are summarized in Table 1. The patients who died in hospital were more likely to be women (42.6% vs. 27.6%, respectively; *P* = 0.04), had a longer total ischemic time (minutes) (390 (237.5, 531) vs. 264.5 (153.75, 359.25), respectively; *P* < 0.001), and had a higher chance of risk factors including no-reflow and type B2–C lesions.

### 3.2. Nomogram Construction

**Figure 1:**
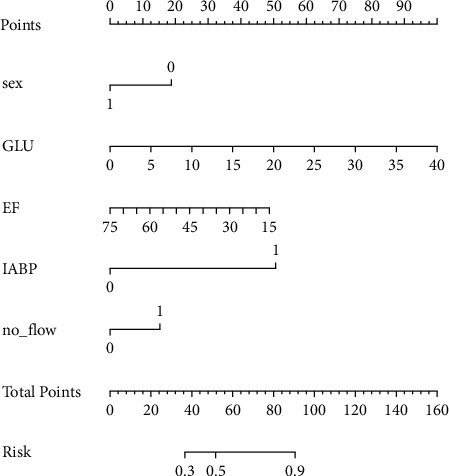
Nomogram for the prediction of in-hospital mortality in patients with acute ST-elevation myocardial infarction after primary PCI. GLU indicates random blood glucose on admission; EF indicates ejection fraction after PCI; IABP indicates intra-aortic balloon pump.

According to multivariate logistic analysis, the five variables met the threshold of *P* < 0.05. Sex (odds ratio (OR) = 3.69, 95% confidence interval (95%CI) = 1.06–1.50; *P* = 0.047), random blood glucose on admission (OR = 1.20, 95%CI = 1.11–1.32; *P* < 0.001), EF after PCI (OR = 0.96, 95%CI = 0.93–0.99; *P* = 0.01), no-reflow grade (OR = 3.11, 95%CI = 1.22–8.27; *P* = 0.01), and IABP (OR = 3.01, 95%CI = 4.50–6.22; *P* = 0.003) were independently associated with in-hospital mortality after PCI in CS-STEMI patients (Table 2). The nomogram is shown in Figure 1. The formula for calculating the total points of the nomogram was 1.8260 + 1.3061 × sex + 0.1888 × random blood glucose on admission −0.0387 × LVEF after PCI + 1.1332 × no-reflow + 3.4047 × IABP.

### 3.3. Evaluation of the Nomogram

**Figure 2:**
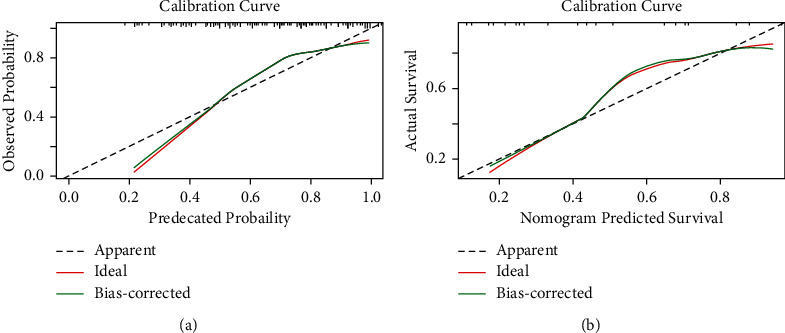
Calibration curves of the nomogram for the training set (a) and the testing set (b).

**Figure 3:**
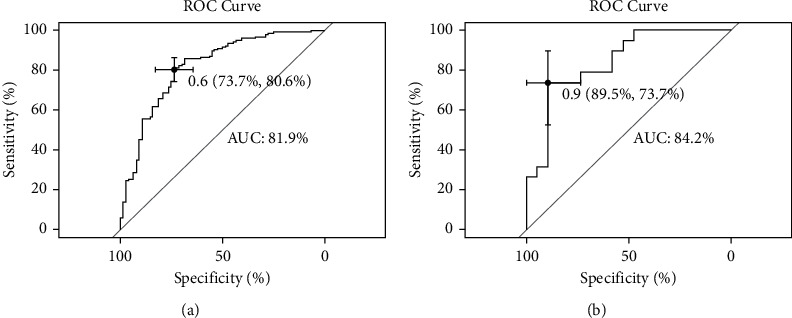
Received operating characteristics (ROC) curves of the nomogram for the training set (a) and the testing set (b).

**Figure 4:**
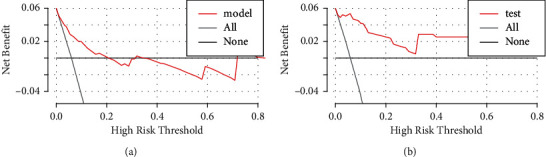
Decision curve analysis (DCA) for the risk model for the training set (a) and the testing set (b).

In the training set, the C-index was 0.819, indicating that the prediction model was valuable in clinical practice (Figure 2(a)). The goodness-of-fit was 0.08, indicating a good prediction accuracy. The ROC curve is shown in Figure 3(a) (AUC = 0.819, 95%CI = 0.759–0.879). The DCA curve for the training set is shown in Figure 4(a), which indicates that the nomogram had a high overall net benefit in predicting in-hospital mortality after PCI treatment.

In the testing set, the C-index was 0.842. The calibration curve is shown in Figure 2(b), indicating a goodness-of-fit of 0.585. The ROC curve is shown in Figure 3(b) (AUC = 0.842, 95%CI = 0.715–0.970). The DCA curve is shown in Figure 4(b). The results of the testing set indicated that the nomogram had good discrimination and a good prediction accuracy and provided a good net benefit.

## 4. Discussion

Despite considerable recent advances in the treatment of STEMI, morbidity and mortality remain unacceptably high in CS-STEMI patients. In the present study, we constructed a relatively accurate clinical nomogram that demonstrated adequate discrimination and calibration power to provide an individualized estimation for in-hospital mortality in CS-STEMI patients after PCI. To construct the nomogram, we used five significant predictors (sex, random blood glucose at admission, EF after PCI, IABP, and no flow).

We found that female sex was an independent factor and had a high in-hospital mortality compared with males. Recent studies have shown a lower rate of early PCI in women and consequently a higher mortality [14]. Women often have atypical complaints of angina pectoris, which results in a time delay from symptom onset to hospitalization and the use of PCI. Furthermore, women often have a worse vascular condition, which is frequently associated with the high comorbidities of diabetes and hypertension. Additionally, cardiovascular disease remains the main cause of death for women. Thus, it is important to develop measures that specifically target women to raise awareness of cardiovascular diseases, allowing early recognition of AMI symptoms and timely medical attention. Nevertheless, clinical judgment remains critical for female CS-STEMI patients [15].

Hyperglycemia at admission will affect the in-hospital outcome, regardless of whether the patient has diabetes (Table 1). A possible mechanism involves an increase in intercellular adhesion molecule-1 associated with hyperglycemia, which increases leukocyte plugging of capillaries, augments platelet-dependent thrombus formation, induces further electrophysiologic alterations, and increases the risk of fatal arrhythmias [16]. Furthermore, hyperglycemia frequently occurs in critically ill patients, such as those with left main disease and multivessel disease [17]. Several studies have reported that high admission blood glucose levels are common after AMI and are associated with an increased risk of death [18, 19], which may relate to more prominent gluconeogenesis from acute stress. Yang et al. also found that blood glucose at admission can increase the accuracy of the GRACE and TIMI risk score models in nondiabetic patients but not in diabetic patients [20].

Since the introduction of aspirin and thrombolytic therapy, LVEF is the most consistent predictor of mortality in STEMI [21] and is included in mortality score systems such as ACEF (age, creatinine, and ejection fraction) [22]. Impaired LV systolic function also remains a strong predictor of cardiovascular events in STEMI patients undergoing PCI [23]. In accordance with previous studies, we found that lower LVEF after PCI was an independent risk factor of in-hospital mortality in CS-STEMI patients, with a significant increase in the odds for in-hospital death for each 1% decrease in LVEF. Current guidelines recommend evaluation of LVEF indicated before discharge [24]. Therefore, echocardiogram with evaluation and treatment of complications to identify patients at a high risk of death during hospitalization is critical.

In the present study, the incidence of no-reflow was 39%. The manifestations of CS were reported to be strongly correlated with development of no-reflow, while patients with no reflow during PCI had difficulty achieving a normal epicardial flow at the end of surgery [25]. The SHOCK trial demonstrated a doubling in the death rate of CS patients with unsuccessful PCI compared with successful PCI [26]. In agreement with previous studies, we found that STEMI patients complicated with CS and those who developed no-flow had increased mortality during overall hospitalization. The mechanism of no-reflow is complicated. Nevertheless, in reperfusion-related injury [27], large numbers of neutrophils and platelets can infiltrate the microcirculation during reperfusion. Furthermore, stent damage to the vessel wall during PCI, resulting in high lipid release into the coronary artery, increased local vascular microthrombosis, and disruption of distal coronary artery microcirculation during PCI may be the main trigger of severe microvascular dysfunction. Ischemia-related injury is also an important factor. Additionally, individual sensitivity can play a role [28].

IABP is the most common mechanical system used for hemodynamic support in CS patients. However, current studies have not shown benefits of IABP on mortality. Three large, randomized studies (IABP SHOCK II, CRISP-AMI, and BCIS-1) have assessed IABP under different conditions. IABP SHOCK II reported no differences in 12-month and 6-year mortality with the use of IABP [29, 30]. Furthermore, IABP did not reduce mortality in CS patients with anterior STEMI or severe ischemic cardiomyopathy [31, 32]. Thus, IABP, as an important adjunct to PCI, does not seem to improve mortality outcomes. This may be because more unstable patients are treated with IABPs and the coronary anatomy of CS patients is more complicated (e.g., left main disease, multivessel disease, or long-lesions disease). Currently, the most commonly used circulatory support devices for STEMI-CS patients are the IABP and Impella devices (intravascular microaxial left ventricular assist devices (LVADs)). Although LVADs improved hemodynamic parameters more than IABP, the existing studies did not show its advantages in reducing mortality and bleeding rates nor did it show overall harm [33]. Studies have shown that early application of ECMO can reduce 30-day mortality, but there are also problems of prolonged door to balloon time and failure of ECMO weaning [34]. This may also be the direction of our future research.

Interestingly, Takotsubo syndrome can also cause a cardiogenic shock. It is primarily preceded by emotional triggers and usually presents with chest pain and difficulty breathing. A possible mechanism is dynamic LVOT occlusion prior to the ischemic event. Once present, dynamic obstruction increases left ventricular filling pressure, increasing myocardial oxygen demand in the central apical cavity. If this condition persists, apical hypoperfusion and ischemia may worsen, eventually leading to apical infarction. At this time, it is necessary to timely diagnose and quickly relieve the obstruction and cardiogenic shock can be relieved [35]. Therefore, time is heart, time is life. In any case, it is necessary to shorten the total ischemic time as much as possible and restore myocardial blood circulation as soon as possible.

In our study, there are 120 (51.9%) patients with multivessel CAD in the training set and 16 (37.2%) patients in the testing set. In fact, almost all patients have only undergone revascularization of the culprit vessels. Surviving patients with multivessel CAD will undergo non-culprit disease PCI within 15–45 days after the first PCI on a voluntary basis. We did not include patients who were readmitted to the hospital in this study. The 2017 ESC guidelines [12] recommend revascularization of non-culprit vessels before discharge for patients with a multivessel disease. However, they did not include patients with a cardiogenic shock, leaving a gap of evidence. In the CULPRIT SHOCK trial [36], the investigators observed that in STEMI-CS patients, revascularization of the culprit vessel alone reduced all-cause mortality and incidence of renal insufficiency within 30 days, possibly related to increased contrast agent dosage and fluoroscopy time, which increased inflammatory activity and had harmful effects on the heart muscle. In the future, we may develop related randomized trials to discuss the impact of complete and incomplete revascularization on the in-hospital mortality of STEMI patients with CS after PCI.

A nomogram is a simple and intuitive representation of a mathematical model that allows for calculating clinical scores [37]. The applicability of the nomogram is that it can reflect the predicted probability of disease recurrence or death into a numerical probability. In addition, to be of clinical usefulness in a routine setting, the nomogram must contain variables assessed in the routine clinical setting. The total score for variables entered into the nomogram corresponds to the predicted probability. Its advantage is the simplicity and ease of use, helping doctors predict individualized risk of each patient. Clinicians can stratify patients based on probability and develop individualized treatment plans. However, despite the increasing use of nomograms, the prospective study is lacking. The question of how to serve clinical better remains to be further explored [38].

Some study limitations should be mentioned. 1. This study has limitations that are inherent to retrospective observational studies. Many hospitals and doctors are involved, which can lead to some missing information, such as electrolyte disorders pre-PCI, adenosine use during PCI, renal failure after PCI; 2. As the ischemic time is shortened as much as possible, patients whose symptoms and/or ECG can be diagnosed are directly treated with PCI. Therefore, other potential risk factors in our study, such as LVEF before PCI, could not be included in the analyses. Further studies are still necessary to confirm the performance of the clinical nomogram in future investigations.

## 5. Conclusions

In conclusion, a nomogram to predict in-hospital mortality in STEMI patients with CS after PCI was developed and validated in Hebei, China. The nomogram showed a satisfactory performance, with a C-index of 0.832. Thus, this nomogram might be a precisely individualized predictive tool for prognosis. Still, additional studies are needed to determine whether it can be applied to other populations before its implementation in clinical practice.

## Figures and Tables

**Table 1:** Clinical characteristics selected as predictors for the nomogram.

Variables	Training set			Testing set		
	Survival (*n* = 76)	In-hospital mortality (*n* = 155)	*P*	Survival (*n* = 19)	In-hospital mortality (*n* = 24)	*P*
Male (*n* (%))	55 (72.4)	89 (57.4)	0.04	14 (73.7)	10 (41.7)	0.073
Total ischemic time (min (median (IQR)))	264.5 (153.75, 359.25)	390 (237.5, 531)	<0.001	247 (142.5,332.5)	282.5 (213.5,415.25)	0.203
IABP (*n* (%))	1 (1.3)	35 (22.6)	<0.001	1 (5.3)	5 (20.8)	0.308
No-reflow (*n* (%))	20 (26.3)	76 (49.0)	0.002	3 (15.8)	6 (25.0)	0.719
Type B2-C (*n* (%))	34 (44.7)	109 (70.3)	<0.001	9 (47.4)	14 (58.3)	0.683
LM (*n* (%))	9 (11.8)	38 (24.5)	0.038	0 (0.0)	6 (25.0)	0.057
Random blood glucose on admission, mmol/L (median (IQR))	7.45 (5.84, 10.27)	12.92 (9.00, 13.47)	<0.001	6.12 (5.23, 9.47)	13.23 (11.71, 14.05)	<0.001
EF after PCI, (median (IQR))	52 (45, 59)	45 (37, 53)	<0.001	55 (45, 58)	41 (35, 41)	<0.001
Medication list on admission n (%)						
DAPT	76 (100.0)	111 (71.6)	<0.001	18 (94.7)	21 (87.5)	0.417
Ticagrelor	36 (47.4)	64 (41.3)		8 (42.1)	11 (45.8)	
Clopidogrel	40 (52.6)	47 (30.3)		10 (52.6)	10 (41.7)	

IABP: intra-aortic balloon pump; EF: ejection fraction.

**Table 2:** Variables selected as predictors for the nomogram according to the multivariable logistic analysis.

Variables	*P*	OR	95% CI
Sex	0.047	3.77	1.02–13.96
Random blood glucose on admission	<0.001	1.2	1.11–1.32
EF after PCI	0.01	0.96	0.93–0.99
IABP	0.003	30.11	3.13–289.32
No-reflow	0.01	3.11	1.2–8.02

OR: odds ratio; CI: confidence interval; EF: ejection fraction; PCI: percutaneous coronary intervention; IABP: intra-aortic balloon pump.

## Data Availability

The data supporting the results in the current study are available from the corresponding author upon reasonable request.

## Supplementary Materials

Supplementary Materials Supplementary Table 1: The clinical characteristics, including demographic, medical history, angiographic characteristics, and information of cardiac procedures, medications, and biochemical markers.
