# Supplementary material for: Nomogram for Predicting In-Hospital Mortality in Patients with Acute ST-Elevation Myocardial Infarction Complicated by Cardiogenic Shock after Primary Percutaneous Coronary Intervention
Source: J Interv Cardiol. 2022 Mar 12;2022:8994106. doi: 10.1155/2022/8994106 (PMC8934239; doi:10.1155/2022/8994106)
Supplement: Supplementary Materials — Supplementary Table 1: The clinical characteristics, including demographic, medical history, angiographic characteristics, and information of cardiac procedures, medications, and biochemical markers. [file 8994106.f1.docx]

Supplementary Table 1 Clinical characteristics of the patients used to construct the nomogram

| variables | Training set | | | Testing set | | |
| --- | --- | --- | --- | --- | --- | --- |
|  | Survival (n=76) | In-hospital mortality (n=155) | P | Survival (n=19) | In-hospital mortality (n=24) | P |
| Age (years) (median (IQR)) | 66.00(59.75,77.50) | 67.00(57.00,74.00) | 0.801 | 62.00(57.5,73.50) | 77.00(67.75,81.25) | 0.007 |
| Male (n (%)) | 55 (72.4) | 89 (57.4) | 0.04 | 14 (73.7) | 10 (41.7) | 0.073 |
| BMI (kg/m2) (median (IQR)) | 25.34 (23.88, 26.73) | 24.5 (24.5, 25.13) | 0.197 | 25.6 (23.95,26.41) | 24.16 (23.30, 25.46) | 0.156 |
| Cardiac arrest (n (%)) | 6 (7.9) | 15 (9.7) | 0.842 | 2 (10.5) | 2 (8.3) | 0.806 |
| Use of temporary pacemaker before admission (n (%)) | 0 (0.0) | 7 (4.5) | 0.141 | 1 (5.3) | 2 (8.3) | 0.695 |
| Ventilator support before admission (n (%)) | 3 (3.9) | 15(9.7) | 0.206 | 0 (0.0) | 3 (12.5) | 0.32 |
| CPR before admission (n (%)) | 6 (7.9) | 20 (12.9) | 0.363 | 1 (5.3) | 4 (16.7) | 0.497 |
| SBP on admission (median (IQR)) | 90 (80, 110) | 98 (81, 121) | 0.225 | 82 (75, 89) | 81 (72, 104) | 0.616 |
| DBP on admission (median (IQR)) | 63 (52, 72) | 62 (52, 77) | 0.707 | 53 (46, 61) | 54 (45, 71) | 0.75 |
| Heart rate on admission (median (IQR)) | 74 (67, 90) | 85 (66, 104) | 0.112 | 73 (65,88） | 75 (59,92） | 0.759 |
| Fatal arrhythmia before admission (n (%)) | 8 (10.5) | 25 (16.1) | 0.346 | 1 (5.3) | 4 (16.7) | 0.497 |
|  |  |  |  |  |  |  |
| Total ischemic time (min (median (IQR))) | 264.5 (153.75, 359.25) | 390 (237.5, 531) | <0.001 | 247 (142.5,332.5) | 282.5 (213.5,415.25) | 0.203 |
| Killip class 3-4 (n (%)) | 32 (42.1) | 84 (54.2) | 0.113 | 4 (21.1) | 18 (75.0) | 0.001 |
| Past medical history |  |  |  |  |  |  |
| *Hypertension (n (%))* | 27 (35.5) | 74 (47.7) | 0.106 | 9 (47.4) | 13 (54.2) | 0.892 |
| *DM (n (%))* | 23 (30.3) | 55 (35.5) | 0.522 | 4 (21.1) | 10 (41.7) | 0.269 |
| *Hyperlipidemia (n (%))* | 34 (44.7) | 58 (37.4) | 0.355 | 8 (42.1) | 13 (54.2) | 0.632 |
| *Previous PCI (n (%))* | 4 (5.3) | 9 (5.8) | 0.866 | 1 (5.3) | 2 (8.3) | 0.695 |
| *Previous CABG (n (%))* | 0 | 2 (1.3) | 0.811 | 0 (0.0) | 1 (5.3) | 0.368 |
| *CAD (n (%))* | 11 (14.5) | 32 (20.6) | 0.341 | 11 (57.9) | 14 (58.3) | 0.977 |
| *AF (n (%))* | 3 (3.9) | 18 (11.6) | 0.097 | 0 (0.0) | 2 (8.3) | 0.198 |
| *HF (n (%))* | 3 (3.9) | 12 (7.7) | 0.415 | 0 (0.0) | 1 (5.3) | 0.368 |
| *Renal insufficiency (n (%))* | 20 (26.3) | 55 (35.5) | 0.212 | 7 (36.8) | 12 (50.0) | 0.58 |
| *History of cerebrovascular disease (n (%))* | 7 (9.2) | 19 (12.3) | 0.64 | 1 (5.3) | 3 (12.5) | 0.777 |
| *Peripheral vascular disease (n (%))* | 1 (1.3) | 3 (1.9) | 0.734 | 0 (0.0) | 1 (5.3) | 0.368 |
| *History of bleeding (n (%))* | 3 (3.9) | 10 (6.5) | 0.637 | 0 (0.0) | 1 (5.3) | 0.368 |
| *Family history of CAD (n (%))* | 4 (5.3) | 10 (6.5) | 0.95 | 3 (15.8) | 4 (16.7) | 0.938 |
| Angiographic characteristics |  |  |  |  |  |  |
| *Long lesions (n (%))* | 29 (38.2) | 79 (51.0) | 0.09 | 11 (57.9) | 13 (54.2) | 0.807 |
| *Thrombus aspiration (n (%))* | 19 (25.0) | 37 (23.9) | 0.98 | 12 (63.2) | 13 (54.2) | 0.553 |
| *Residual stenosis (n (%))* | 2 (2.6) | 11 (7.1) | 0.28 | 0 (0.0) | 4 (16.7) | 0.062 |
| *Use temporary pacemaker (n (%))* | 4 (5.3) | 22 (14.2) | 0.072 | 0 (0.0) | 3 (12.5) | 0.32 |
| *IABP (n (%))* | 1 (1.3) | 35 (22.6) | <0.001 | 1 (5.3) | 5 (20.8) | 0.308 |
| *Respirator support (n (%))* | 5 (6.6) | 23 (14.8) | 0.111 | 1 (5.3) | 5 (20.8) | 0.308 |
| *Pericardial aspiration (n (%))* | 0 (0.0) | 4 (2.6) | 0.381 | 0 (0.0) | 2 (8.3) | 0.576 |
| *No-reflow (n (%))* | 20 (26.3) | 76 (49.0) | 0.002 | 3 (15.8) | 6 (25.0) | 0.719 |
| *Dissection (n (%))* | 0 (0.0) | 2 (1.3) | 0.811 | 0 (0.0) | 1 (4.2) | 0.368 |
| *Acute HF (n (%))* | 15 (19.7) | 40 (25.8) | 0.394 | 2 (10.5) | 6 (25.0) | 0.414 |
| *Bleeding (n (%))* | 0 (0.0) | 6 (3.9) | 0.194 | 0 (0.0) | 2 (8.3) | 0.576 |
| *Type B2-C (n (%))* | 34 (44.7) | 109 (70.3) | <0.001 | 9 (47.4) | 14 (58.3) | 0.683 |
| *TIMI flow grade 0-1 before PCI (n (%))* | 38 (50.0) | 99 (63.9) | 0.061 | 15 (78.9) | 19 (79.2) | 0.986 |
| *Use of GP IIb/IIIa inhibitors* ***(n (%))*** | 46 (29.7) | 16 (21.1) | 0.165 | 6 (25.0) | 4 (21.1) | 0.761 |
| *LM (n (%))* | 9 (11.8) | 38 (24.5) | 0.038 | 0 (0.0) | 6 (25.0) | 0.057 |
| *LAD (n (%))* | 17 (22.4) | 54 (34.8) | 0.075 | 5 (26.3) | 12 (50.0) | 0.206 |
| *Multivessel CAD (n (%))* | 36 (47.4) | 84 (54.2) | 0.404 | 5 (26.3) | 11 (45.8) | 0.189 |
| Biochemical markers |  |  |  |  |  |  |
| *Anemia (n (%))* | 6 (7.9) | 20 (12.9) | 0.363 | 2 (10.5) | 5 (20.8) | 0.622 |
| *N/L ratio (median (IQR))* | 5.02 (2.76, 8.49) | 9.19 (3.32, 9.88) | 0.078 | 7.61 (4.9, 14.16) | 10.26 (7.37, 13.89) | 0.695 |
| *HCT, % (median (IQR))* | 40.1 (37.05, 42.9) | 38.96 (37.35, 42.3) | 0.163 | 39.4 (36.65, 42.3) | 37.45 (33.88, 44.20) | 0.334 |
| *PLT, ×109/L (median (IQR))* | 228 (194.0, 264.0) | 245 (207, 272) | 0.132 | 232 (176, 254) | 192 (183, 235) | 0.163 |
| *Random blood glucose on admission, mmol/L (median (IQR))* | 7.45 (5.84, 10.27) | 12.92 (9.00, 13.47) | <0.001 | 6.12 (5.23, 9.47) | 13.23 (11.71, 14.05) | <0.001 |
| EF after PCI, (median (IQR)) | 52 (45, 59) | 45 (37, 53) | <0.001 | 55 (45, 58) | 41 (35, 41) | <0.001 |
| Medication list on admission n(%) |  |  |  |  |  |  |
| *DAPT* | 76 (100.0) | 111 (71.6) | <0.001 | 18 (94.7) | 21 (87.5) | 0.417 |
| *Ticagrelor* | 36 (47.4) | 64 (41.3) |  | 8 (42.1) | 11 (45.8) |  |
| *clopidogrel* | 40 (52.6) | 47 (30.3) |  | 10 (52.6) | 10 (41.7) |  |
| *ACEI/ARB* | 4 (5.3) | 6 (3.9) | 0.885 | 1 (5.3) | 5(20.8) | 0.308 |
| *β-Blocker* | 1 (1.3) | 6 (3.9) | 0.512 | 1 (5.3) | 3 (12.5) | 0.777 |
| *Statin* | 4 (5.3) | 14 (9.0) | 0.458 | 2 (10.5) | 3 (12.5) | 0.841 |

BMI: body mass index; SBP: systolic blood pressure; DBP: diastolic blood pressure; DM: diabetes mellitus; PCI: percutaneous coronary intervention; CABG: coronary artery bypass graft; CAD: coronary atherosclerotic heart disease; AF: atrial fibrillation; HF: heart failure; IABP: intra-aortic balloon pump; MI: myocardial infarction; LAD: left anterior descending branch; N/L ratio: neutrophils/lymphocytes ratio; HCT: hematocrit; HGB: hemoglobin; PLT: platelets; EF: ejection fraction; ACEI: angiotensin-converting enzyme inhibitor; ARB: angiotensin receptor blocker.
